# From practice to lecture hall: Optimizing communication courses in medical education

**DOI:** 10.3205/zma001773

**Published:** 2025-09-15

**Authors:** Friederike Laudage, Thomas Kötter, Daniel Wiswede

**Affiliations:** 1 University Medical Centre Schleswig-Holstein, Lübeck Campus, Institute of Medical Psychology, Lübeck, Germany; 2 University Medical Centre Schleswig-Holstein, Lübeck Campus, Institute of General Medicine, Lübeck, Germany

**Keywords:** communication skills, medical education, doctor-patient communication, communication training, National Competency-based Learning Objectives Catalogue for Medicine (NKLM)

## Abstract

**Objective:**

Communication skills are a central component of the medical profession and are already taught during medical studies. However, the design of teaching content for doctor-patient communication across Germany is not based on empirical data collected from physicians’ everyday professional practice, meaning optimal preparation for future work is not guaranteed. The aim of this study was to identify the need for relevant topic content and to use the limited teaching time in communication courses for these topics, providing educators with empirically based selection criteria.

**Methodology:**

Using an exploratory sequential mixed-methods design, the need for relevant topics was determined. All teaching practices of the University of Lübeck were invited in writing to participate in the study. Teaching physicians rated, using a topic catalogue, which topics they considered relevant for communication courses in medical studies, as well as how challenging and frequent these topics are in everyday professional life.

**Results:**

The questionnaire was completed by 46 of the 70 invited teaching physicians (65.7%). Fifteen topics, including *breaking bad news* and *communicating one’s own mistakes*, were rated as relevant for communication courses.

**Conclusion:**

The results provide tailored recommendations for case studies in communication courses that are relevant for medical students’ later professional practice. A concrete implementation involves the selection of relevant topics, enabling the practice of frequently encountered but less challenging topics at the beginning of studies and more difficult but rarer topics in advanced studies.

## Zusammenfassung

**Zielsetzung:**

Kommunikative Fertigkeiten sind zentraler Bestandteil des Arztberufs und werden bereits im Medizinstudium geschult. Die Ausgestaltung der Lehrinhalte für die Arzt-Patienten-Kommunikation basiert deutschlandweit jedoch nicht auf empirisch erhobenen Daten aus dem Berufsalltag von Ärztinnen und Ärzten, weshalb eine optimale Vorbereitung auf die spätere Tätigkeit nicht sichergestellt ist. Ziel dieser Studie war, den Bedarf an relevanten Themeninhalten zu identifizieren und die knappe Unterrichtszeit der Kommunikationskurse für diese zu verwenden und Lehrenden die empirisch basierten Auswahlkriterien zur Verfügung zu stellen.

**Methodik:**

Mittels eines explorativ-sequentiellen Mixed-Methods-Designs wurde der Bedarf an relevanten Themen ermittelt. Alle Lehrpraxen der Universität zu Lübeck wurden schriftlich zur Studienteilnahme eingeladen. Die Lehrärztinnen und Lehrärzte gaben in einer Ratingskala mithilfe eines Themenkatalogs an, welche Themen als relevant für Kommunikationskurse im Medizinstudium erachtet werden und wie herausfordernd und häufig diese Themen im Berufsalltag sind.

**Ergebnisse:**

Der Fragebogen wurde von 46 der 70 angeschriebenen Lehrärzt:innen (65,7%) ausgefüllt. Fünfzehn Themen, darunter das *Überbringen schlechter Nachrichten* und die *Kommunikation von eigenen Fehlern*, wurden als relevant für Kommunikationskurse eingeschätzt.

**Schlussfolgerung:**

Die Ergebnisse liefern passgenaue Handlungsempfehlungen für Fallbeispiele in Kommunikationskursen, die im späteren Berufsalltag der Medizinstudierenden relevant sind. Eine konkrete Umsetzung umfasst die Auswahl an relevanten Themen, wodurch zu Beginn des Studiums im ärztlichen Alltag häufig behandelte, aber als wenig herausfordernde angesehene Themen und im fortgeschrittenen Studium schwierige, aber seltene Themen geübt werden können.

## 1. Introduction

### 1.1. Importance of communication skills in medicine

Communication skills are central competencies [1], that at are essential for the doctor-patient relationship [2] and effective medical treatment [3], [4], [5]. Studies have already demonstrated numerous positive effects of communication skills regarding the doctor-patient relationship [2], therapy adherence [6], patient satisfaction [7], [8] and treatment success [9]. On the part of physicians, good communication skills are associated with lower emotional exhaustion, increased self-efficacy [10], and higher job satisfaction [8]. In contrast, a lack of communication skills can lead to unstructured conversations, interruptions of patients, and unclear information regarding diagnosis and therapy [11], often resulting in patients being unable to recall therapy recommendations after the medical consultation [12]. Deficits in communication contribute to increased medical errors [13] and a higher number of complaints and lawsuits against physicians [14]. It is now undisputed that effective communication within the doctor-patient relationship can be learned [15], [16], [17] and therefore is already taught during medical studies, with scientific evidence supporting the value of longitudinal curricula [18], [19].

### 1.2. Teaching in medical psychology

Following the state of research regarding the learnability and teachability of communication skills, medical school curricula have been continuously revised in recent years [20]. In this context, learning objective catalogues are widely used in the implementation and assessment of clinical competencies and are considered quality standards for the execution of communication curricula [21]. In addition to learning objective catalogue specialized in communication skills [21], [22], [23], [24], the German National Competence-Based Learning Objective Catalogue for Medicine (NKLM) [https://nklm.de/menu/] and the catalogues of exam-relevant topics (GK) serve as the foundation for the competencies to be acquired in medical studies, thereby facilitating the implementation of the medical licensure act [20]. The learning objectives of the GK are exam-relevant for medical students and therefore binding. In contrast, the NKLM currently has a recommendatory character but will also become mandatory in medical education in the future [25]. As part of the curriculum reform, the content is to be further developed [26], having medical faculties compare the existing curricula with the NKLM [25] and adjust them accordingly. In this process, teaching should increasingly focus on conveying physician-related competencies, establishing a closer connection to practical applications, and thereby improving and strengthening doctor-patient communication [27]. 

However, immense scope of the learning objectives presents significant challenges [28]. The NKLM and GK aim to include typical application contexts with over a hundred learning objectives. However, the large number of objectives complicates the selection of suitable case examples for communication courses. Although lecturers view the NKLM positively and are willing to use it in teaching, nearly half find its application cumbersome and challenging [25]. Therefore, practicality and the selection of suitable application examples are the greatest challenges in implementing the NKLM in accordance with political requirements. 

### 1.3. Teaching communicative competencies

In a previous study, physicians were asked to retrospectively evaluate their medical education in order to provide guidance for improving medical teaching [29]. Primarily, deficits were identified regarding communication skills, particularly in their instruction. Specifically, the limited number of courses aimed at improving communication skills and the lack of practical application in medical studies were criticized. While 85% of aspiring physicians described communication skills as a foundation for their professional work, only slightly more than a third felt well-prepared for clinical practice. 

In a recent study, physicians and medical students were surveyed for the first time about which topics and learning objectives of the current medical curriculum (GK) are relevant to medical practice [27]. The results show that for both students and physicians, the most relevant topics were those directly related to doctor-patient communication. However, no subchapters or individual topics of the GK were evaluated separately, and no differentiated assessment of the various topics could be conducted. 

Due to the limited time resources available at universities for communication courses, it is not possible to teach all topics outlined in the GK or NKLM. How to select appropriate learning objectives remains unclear, as does the prioritization of individual case studies. Additionally, it is uncertain whether certain topics are more appropriate for introduction at the beginning of a course or in an advanced stage. These uncertainties form the research questions and the associated aim of this study, which focuses on identifying the need for relevant topics in communication courses. This need is assessed based on topics relevant to everyday professional practice and is therefore conducted with physicians who have extensive professional experience. Particularly suited for this purpose are general practitioners working as teaching physicians in academic practices.

### 1.4. Objectives and research questions

The aim of this study was to determine the need for relevant topics in communication courses in medical education from the perspective of practicing teaching physicians in Lübeck:

1. Which topics do practicing physicians consider relevant for case studies in communication courses in medical education?
2. Which topics present particular challenges?
3. Which topics occur most frequently in everyday professional practice?

The results of this study can be used to align the objectives of the *Masterplan Medizinstudium 2020* reform with the needs of practicing physicians [27], thereby enabling adjustments to communication courses in medical education. This aims to improve teaching nationwide and, in the long term, enhance the interaction between physicians and patients. 

## 2. Methods

This study is based on an exploratory sequential mixed-methods design. The results of the qualitative preliminary study guided the design of the subsequent follow-up study [30]. Based on semi-structured expert interviews with general practitioners (*N*=4), relevant topics were identified and, following an extended literature review, a questionnaire with an updated topic catalogue was developed. Prior to conducting the main study described below, positive ethics approval was obtained from the Ethics Committee of the University of Lübeck (21-452), as well as for the preliminary study (19-193A).

### 2.1. Participants

Since the development of communication courses requires coordination and cooperation between medical psychology, which teaches communication skills, and general medicine [31], general practitioners were selected as the target group. This choice is justified by the high frequency of patient contact among general practitioners, with more than 800 cases per quarter [32], as well as the broad spectrum of illnesses treated in general medical practices. The needs analysis was conducted with practicing physicians as their professional experience allows them to provide insights into which study content is highly relevant to their daily work [27]. A special significance is attributed to the academic teaching practices. Through their supervision during the block internship and the practical year, they interact with medical students and thus serve as a link between academic studies and clinical professional practice.

### 2.2. Questionnaire as a data collection instrument

The six-page questionnaire included the assessments of the teaching physicians regarding their experiences with specific topics in the field of doctor-patient communication. The questionnaire’s topic catalogue was developed based on a literature review of learning objective catalogues and scientific guidelines [22], [24], [33], [34], [35], [36], [37], academic textbooks [11], [38], [39], [40], a systematic literature review [41], relevant studies [27], [31], [42], [43], [44], [45], [46], [47], [48], [49], [50], and the results of the expert interviews. Topic contents were included in the questionnaire’s topic catalogue if they were mentioned at least twice in the literature and/or expert interviews. The topic catalogue included 38 specific topics, such as taking *medical histories, breaking bad news*, or *addressing addiction issues* (see attachment 1 ). The items in the topic catalogue mainly consisted of closed-ended questions and free-text fields for additional topics not mentioned in the topic catalogue. Using a five-point rating scale, the relevance of individual topics for communication courses in medical education was assessed (1=“irrelevant” to 5=“relevant”). Additionally, the teaching physicians provided information on the level of challenge (1=“very easy” to 5=“very difficult”) and frequency of occurrence (1=“very rare” to 5=“very often”) of each topic in their professional daily routine. The decision to use a five-point scale was based on methodological considerations to ensure sufficient differentiation while maintaining comprehensibility and practicality [51]. Additionally, sociodemographic data (age, gender) and information regarding professional activity (type of practice, specialist qualification, and duration of professional experience), as well as data on continuing education behaviour and preparation for professional practice, were collected. 

### 2.3. Recruitment and conduct of the survey 

All academic teaching practices at the University of Lübeck (convenience sample) were invited to participate by postal mail. Participation was voluntary and could be discontinued at any time without providing reasons. Personal data were pseudonymized. Each practice was contacted by phone one month before the questionnaire was sent to inform them about the study. An email announcing the questionnaire was also sent, as repeated contacts, especially through networks, increase willingness to participate [52]. Additional measures to increase response rates included a personalized cover letter, sending the questionnaire outside of busy work periods, and reminder emails [52]. The postal mailing included the participant information sheet, consent form, questionnaire, and a small incentive. The teaching physicians completed the questionnaire, which had an anticipated completion time of 10-15 minutes based on a pretest. 

### 2.4. Data analysis

The quantitative data from the questionnaire were analysed using Jamovi (version 1.6.23) and R [53], with figures visualized using ggplot2 [54] and Inkscape. Descriptive analysis of nominal data included absolute frequencies and relative frequencies expressed as percentages. For the statistical analysis of the five-point rating scales, means, standard deviations, relative frequencies (percentages), and absolute frequencies were reported. No substantially new topics or themes relevant to the research question were mentioned in the free-text responses. Three questionnaires were excluded from analysis because they were not completed by teaching physicians.

## 3. Results

### 3.1. Demographics, continuing education, and preparation for the profession 

A total of 46 out of 70 invited teaching physicians (response rate: 65.7%) from 33 of the 37 general medicine teaching practices in Lübeck (89.2%) participated in the written survey conducted between January and February 2022. Table 1 (Tab. 1) provides an overview of the sociodemographic data of the participants (*N*=46). The majority of teaching physicians were aged between 51 and 65 years and had more than 10 years of professional experience. Most of them held specialist training in general medicine and internal medicine. Approximately three-quarters (75.6%) of the participants had previously attended a course on doctor-patient communication. Regarding their current work, more than three-quarters (77.8%) felt adequately prepared for communication with patients. 

### 3.2. Relevant topics for communication courses

The surveyed teaching physicians rated a total of fifteen topics as on average “relevant” (mean score of 4.50 or higher) for communication courses in medical education (see table 2 (Tab. 2) and attachment 2 ). The *initial consultation* was rated as the most relevant topic on average for communication courses. 

### 3.3. Level of challenge of topics in professional practice

Only five topics were rated by the teaching physicians as on average “difficult” (mean score 3.5-4.49). *Language barriers* represent the greatest challenge in everyday professional practice (see table 3 (Tab. 3) and attachment 2 ). No topic was rated as “very easy” (mean score 1.50 or lower) or “very difficult” (mean score 4.50 or higher). 

### 3.4. Frequency of topics occurring in professional practice

The analysis of the frequency of occurrence of individual topics in daily professional practice shows that only two topics were rated as “very often” (mean score 4.50 and above). The most frequently occurring topics on average were taking *medical histories* and* chronic illnesses*.

An additional twelve topics were identified as frequently occurring in doctor-patient communication and were therefore rated as “often” on average (mean score 3.50 to 4.49) in professional practice (see table 4 (Tab. 4) and attachment 2 ).

### 3.5. Relevant topics considering challenge and frequency of occurrence

**Figure 1:**
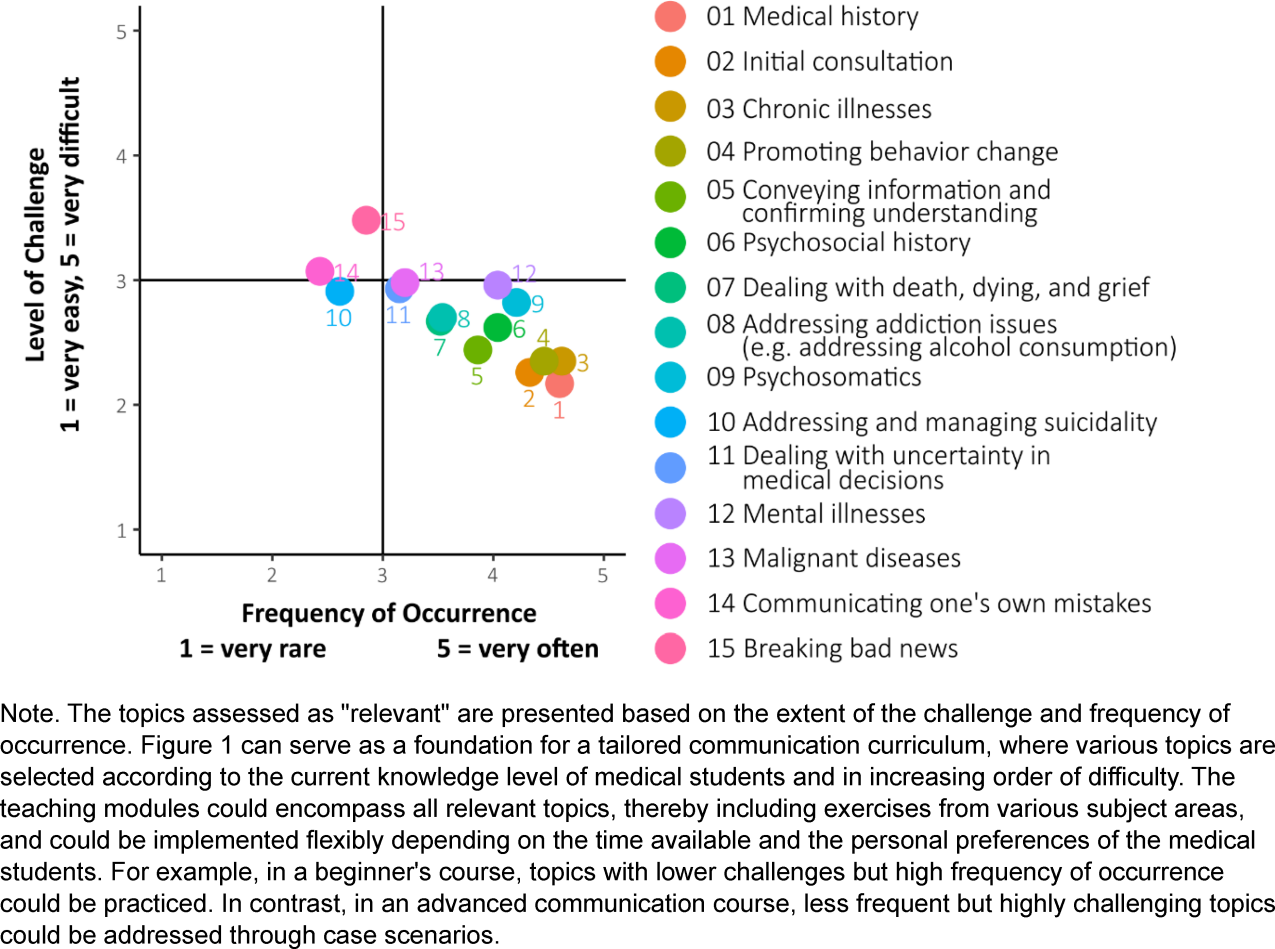
Extent of challenge and frequency of occurrence for relevant topics

Figure 1 (Fig. 1) illustrates how frequently and how challenging the topics rated as relevant for communication courses were assessed. Among the relevant topics, it becomes apparent that difficult topics tend to occur less frequently, whereas frequently occurring topics are generally perceived as less challenging. For example, taking *medical histories* and *chronic illnesses are experienced *as very frequent but easy topics. In contrast,* breaking bad news* and *communicating one’s own mistakes* are the two most challenging relevant topics but occur only occasionally or rarely (see figure 1 (Fig. 1)).

Regarding the level of challenge, none of the relevant topics were rated as very easy or very difficult. In terms of frequency, none of the relevant topics were rated as rare or very rare.

**Figure 2:**
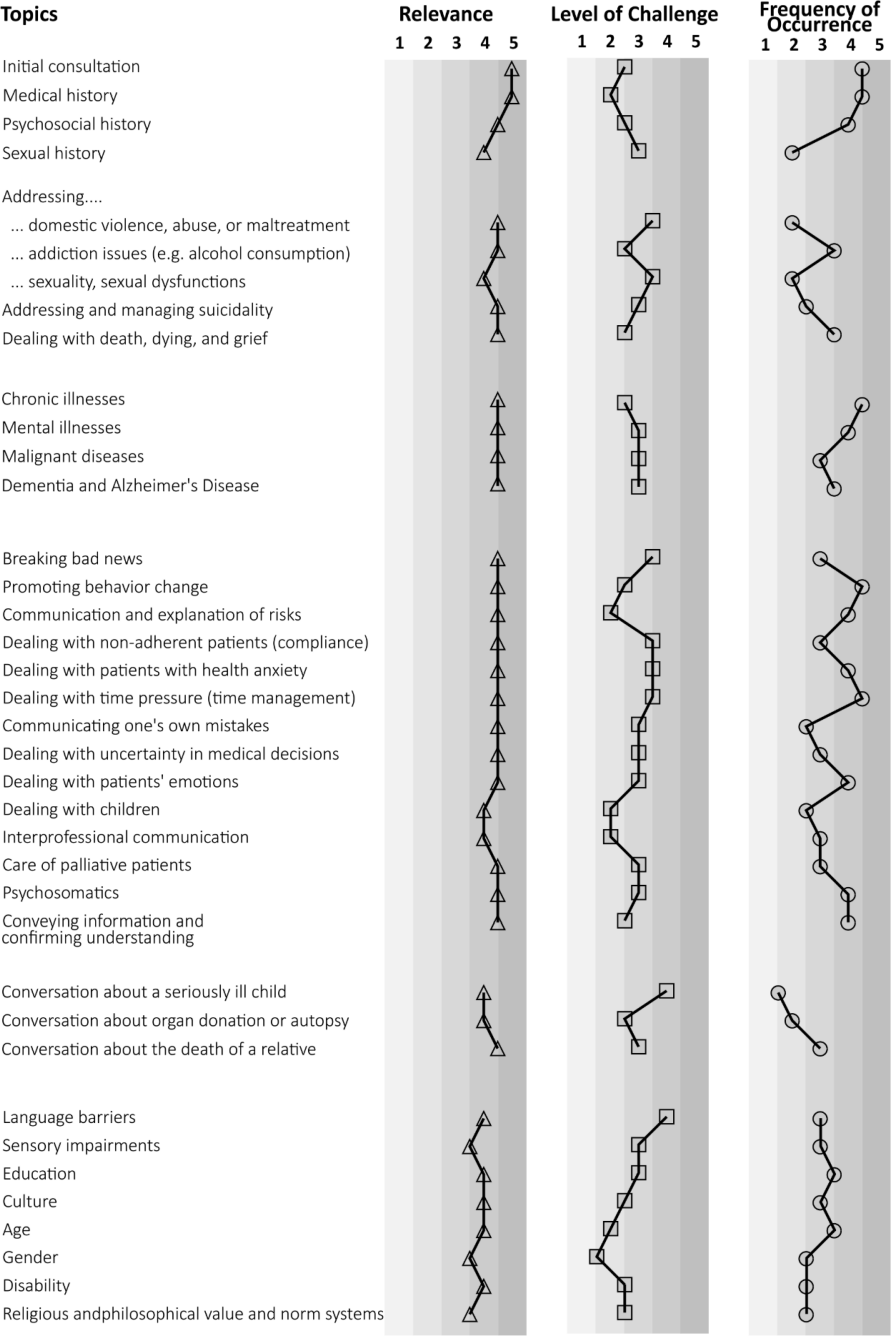
Graphical representation of rating scales Note: The tabular representation includes all 38 topics from the topic catalogue, along with the assessments provided by teaching physicians. For better clarity, the mean values are scaled in increments of 0.5. a Relevance: 1=irrelevant, 2=somewhat irrelevant, 3=neither, 4=somewhat relevant, 5=relevant b Challenge: 1=very easy, 2=easy, 3=neither, 4=difficult, 5=very difficult c Frequency: 1=very rare, 2=rare, 3=occasional, 4=often, 5=very often

Figure 2 (Fig. 2) graphically presents the mean values of all topics using a rating scale to provide an overview of all 38 assessed topics.

## 4. Discussion

This study identified the need for relevant topic content within doctor-patient communication, as well as the level of challenge and frequency of occurrence of the respective topics. The results support the tailored design of teaching modules for communication courses in medical education and provide concrete recommendations for case studies in communication courses that are applicable during medical studies and relevant in later professional practice.

Learning objectives from the scope of the NKLM and GK can now be more easily selected and prioritized for teaching as needed. This significantly simplifies and clarifies the often-criticized handling of learning objective catalogues.

In the present study, the varying mean values within the rating scales indicate that the range of challenge and frequency of occurrence covers a wider spectrum than the mean values of the most relevant topics, which are clustered more closely together and tend to be higher. This suggests a greater consensus among the teaching physicians regarding the relevant topics. The usefulness of capturing these individual aspects is further illustrated by the fact that only five topics were rated as particularly challenging, while fifteen topics were clearly selected as relevant for communication courses. Notably, all challenging topics were rated only as “somewhat relevant” rather than “relevant” for communication courses. This shows that the separate assessments by teaching physicians regarding the relevance of topics, in addition to the level of challenge and frequency of occurrence, are crucial. While clinical relevance is helpful for reducing and selecting learning objectives [55], the present study reveals little differentiation on this basis alone, with many topics remaining since none were rated as “irrelevant”, “somewhat irrelevant”, or “neither”. Only the specification regarding challenge or frequency in everyday professional practice can provide concrete guidance for communication courses.

### 4.1. Limitations

Given the exploratory approach of this study, the results cannot be generalized without further studies. When selecting the target group, it should be considered that the assessments may differ from those of physicians in other specialties. Since the sample consists exclusively of teaching physicians from one university, it is unclear to what extent these results can be transferred to other medical faculties or specialties. Further studies with broader samples would be necessary to examine generalizability. Additionally, the intrinsic motivation to participate in research inquiries may be higher among teaching practices. This is underscored by a significantly higher participation rate among teaching practices compared to practices without a teaching function [56]. This potential selection bias may result in demographic data and study outcomes differing from those of other populations. 

Another limitation is that no existing standardized measurement instrument could be used, as no suitable survey tool was available for the research questions. The newly developed questionnaire was designed specifically for the objectives of this study and created following guidelines for questionnaire construction [51], [57], [58]. However, it was implemented for the first time in this study, therefore quality criteria could not be fully assessed. The categorization was reviewed by several individuals but was not statistically tested for interrater reliability. A formal evaluation would be advisable in future studies to ensure the reproducibility of the categorization. 

### 4.2. Best practice: example of a modified longitudinal communication curriculum 

The study results provide recommendations for topic selection in communication curricula. Table 5 (Tab. 5) presents the Lübeck example of a longitudinal communication curriculum incorporating new topics based on the study findings. The structure of this communication curriculum was designed according to the level of challenge and frequency of occurrence of individual relevant topics in everyday professional practice. In this example, topics assessed as frequent, such as the *initial consultation*, are introduced at the beginning of medical training because they are perceived as relatively easy. Subsequently, topics that are also frequent but increase in difficulty, such as *addressing addiction issues*, are covered. In the advanced phase of the communication course, difficult but less frequent topics, such as* communicating one’s own mistakes* or *dealing with uncertainty in medical decisions*, can be practiced. Additional learning objectives can be flexibly integrated into the respective topic of each session.

## 5. Conclusion

Future doctors should be better prepared for communication with patients to improve the quality of medical care and treatment. Communication courses can now be designed to meet the needs of relevant topics, particularly considering the level of challenge and frequency of occurrence of individual topics in everyday professional practice. The results of this study provide concrete guidance while remaining flexible enough to accommodate the needs of medical students. The high participation rate of teaching physicians underscores the importance of communication skills and the urgent need for further research. However, the results also highlight significant research gaps in the selection of content for communication training. It is crucial that different medical scenarios can be practiced in communication courses and that these contents are systematically selected. While this study does not provide generalizability, it offers initial insights into relevant topics in doctor-patient communication. Further empirical studies on a larger scale and with appropriate samples are necessary to validate the results and explore potential specialty-specific nuances, the perspective of medical students, and, importantly, the view of patients in doctor-patient communication.

## Acknowledgements

The authors would like to sincerely thank all teaching physicians and their practice teams for their support and participation in this study. 

## Authors’ ORCIDs

- Friederike Laudage: [0009-0000-5583-668X]
- Thomas Kötter: [0000-0002-2266-0531]
- Daniel Wiswede: [0000-0002-2702-8316]

## Data

Data for this article are available from Dryad Repository: [https://doi.org/10.5061/dryad.6q573n66x] [59]

## Competing interests

The authors declare that they have no competing interests. 

## Supplementary Material

Questionnaire with topic catalogue

Supplementary material

## Figures and Tables

**Table 1:**
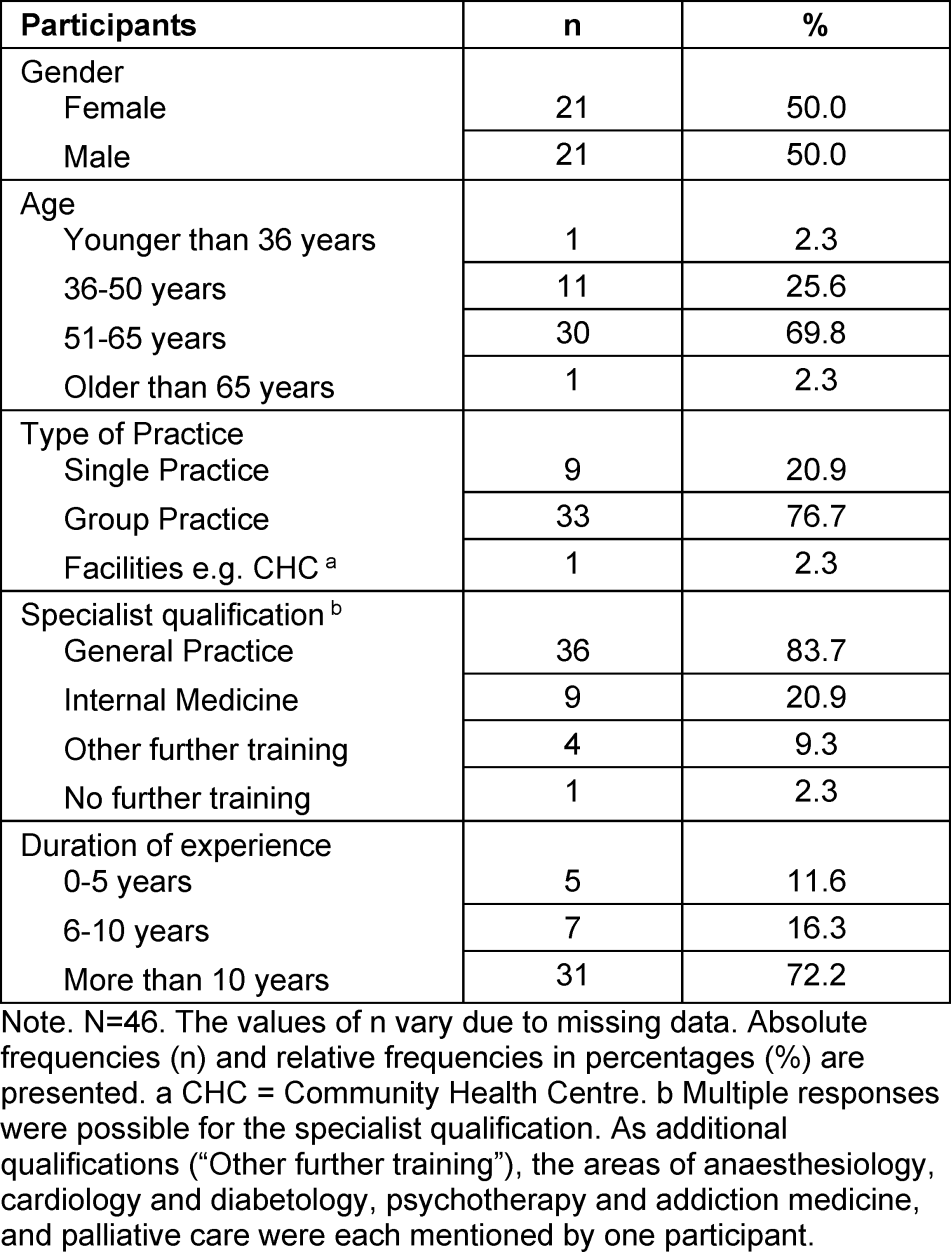
Sociodemographic characteristics of participants

**Table 2:**
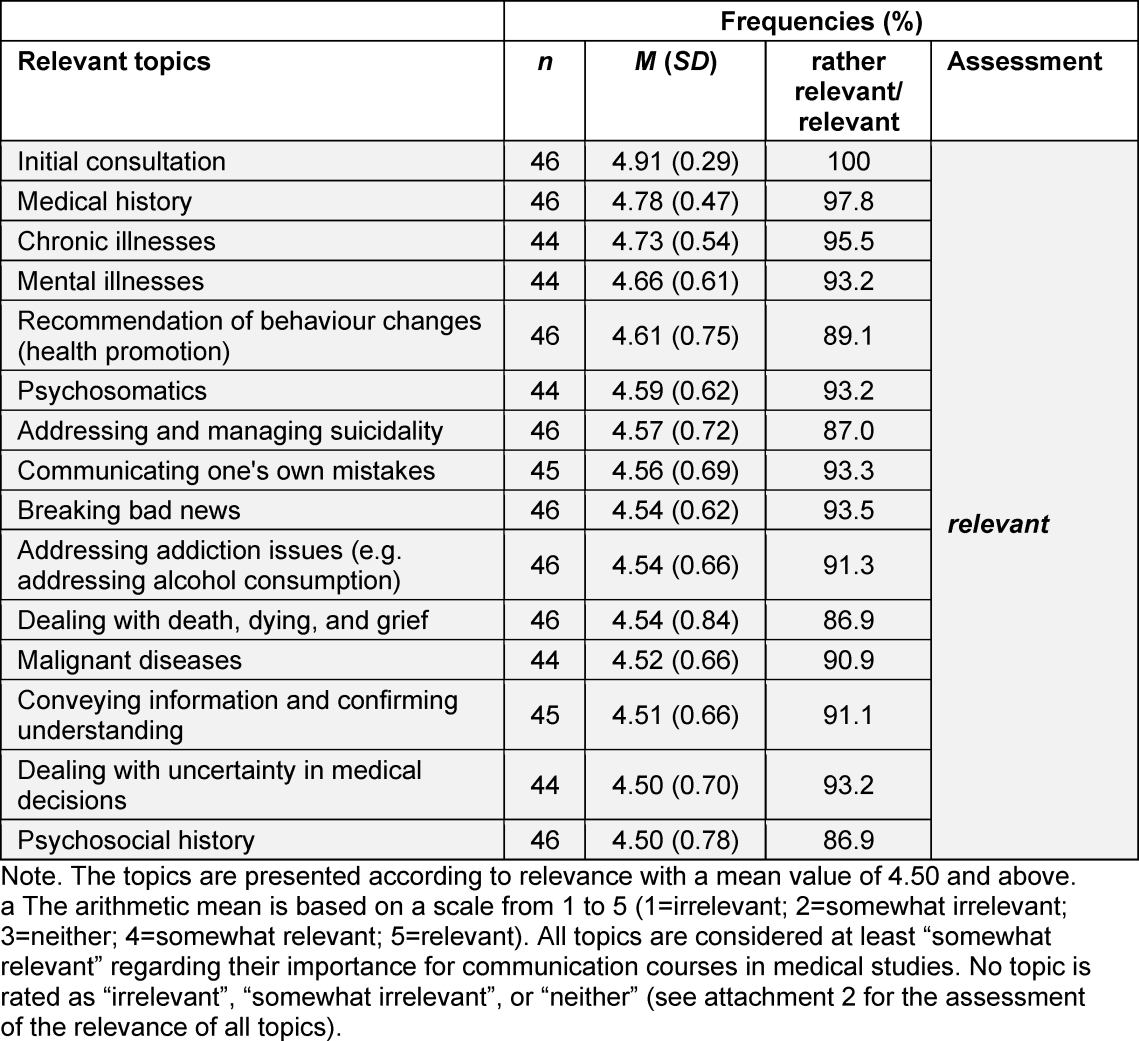
Relevance of individual topics for communication courses

**Table 3:**
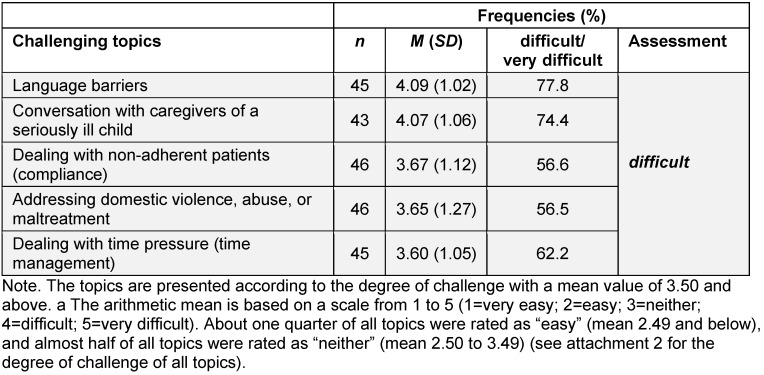
Degree of challenge of individual topics in professional daily practice

**Table 4:**
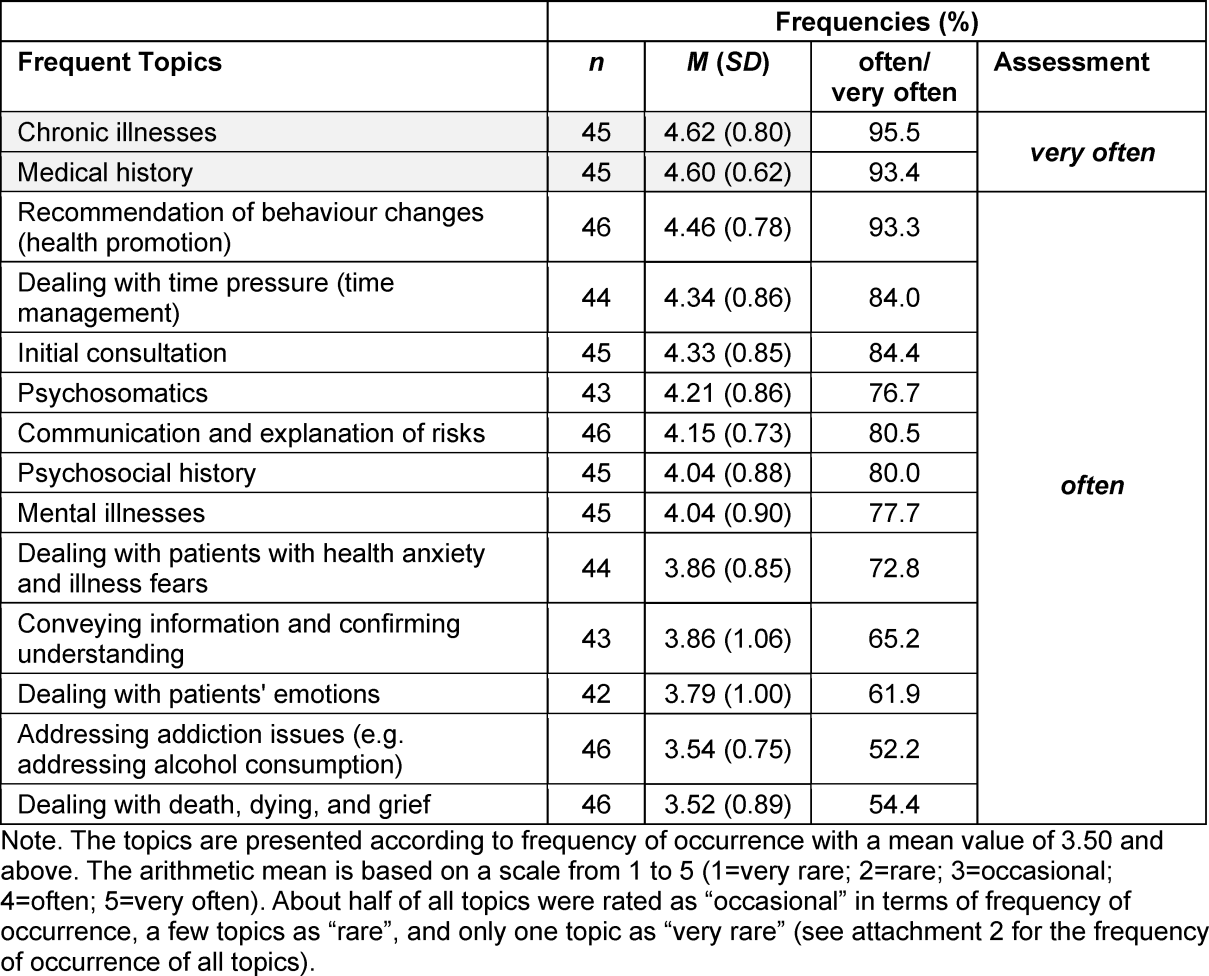
Frequency of occurrence of individual topics in professional daily practice

**Table 5:**
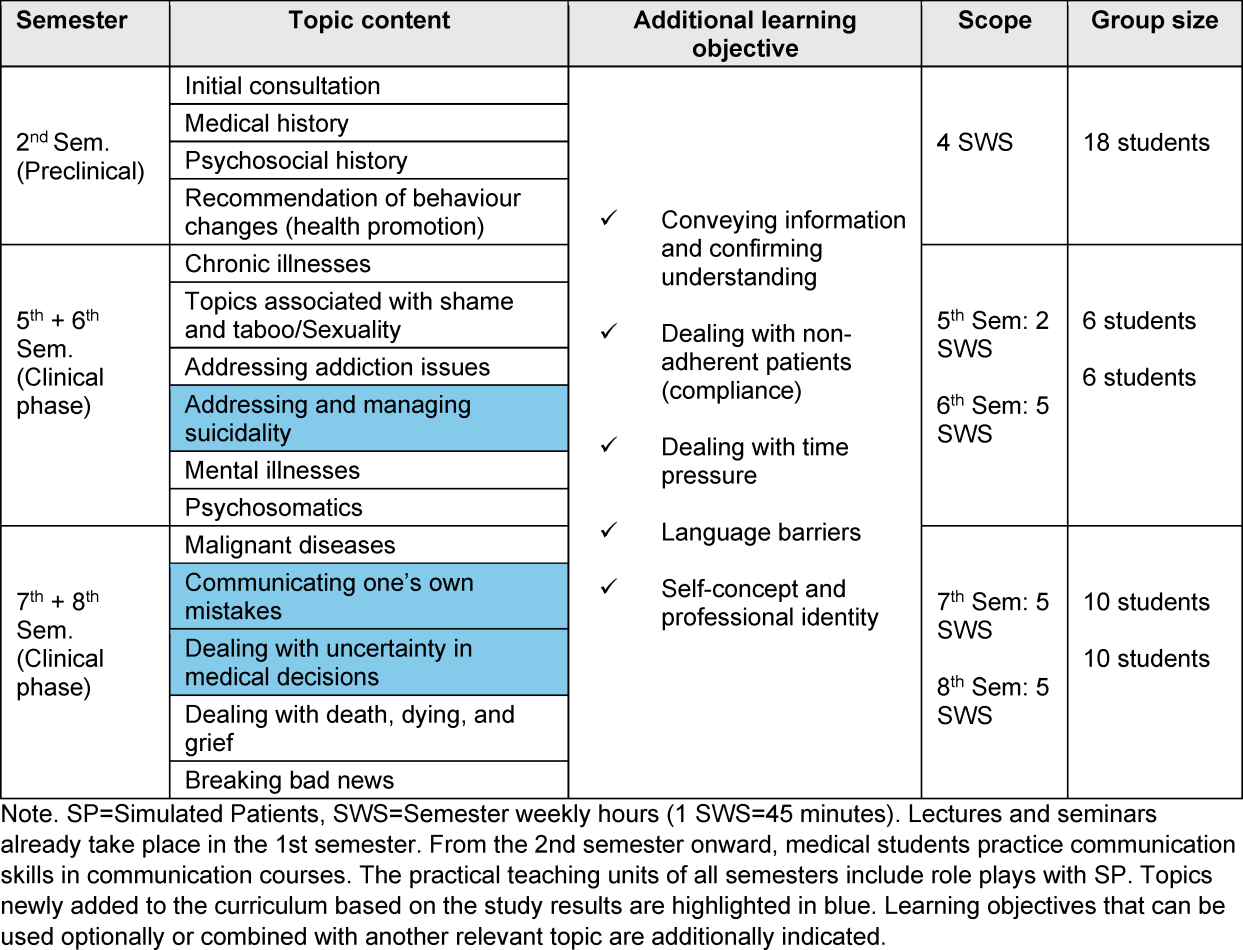
Modified communication curriculum using the Lübeck example
