# Supplementary material for: From practice to lecture hall: Optimizing communication courses in medical education
Source: GMS J Med Educ. 2025 Sep 15;42(4):Doc49. doi: 10.3205/zma001773 (PMC12527397; doi:10.3205/zma001773)
Supplement: Questionnaire with topic catalogue [file JME-42-49-s-001.pdf]

Attachment 1: Questionnaire with topic catalogue

UNIVERSITÄT ZU LÜBECK

Thank you very much for your participation and **valuable support**!

To comprehensively capture the general practitioners' perspective on their experiences in doctor-patient communication within this study, every response is essential. Only in this way can we determine which topics are relevant for inclusion in communication training courses.

Completing the questionnaire will take approximately 10 to 15 minutes.
We kindly request sincere and **prompt responses to the questions**, as there are no right or wrong answers. We are particularly interested in **your personal experiences in general medical practice**. By completing the questionnaire, you are making a significant contribution to improving medical education.

General Notes:
To keep the questionnaire as concise as possible, the masculine form is used throughout. However, all genders are equally implied. The questionnaire number is solely for follow-up purposes, such as reminding participants about returning the form, if necessary. Your data will be stored and analysed electronically without any direct reference to your name.

(A) Personal Experiences in Doctor-Patient Communication

Below you will find a list of various topics related to doctor-patient communication, accompanied by some examples.
Please indicate how **challenging** ("very easy" to "very difficult"), **frequent** ("very rare" to "very often"), and **significant** ("unimportant" to "very important") each topic is in your daily professional communication with patients. Additionally, please assess the **relevance** of each topic for **medical education** ("irrelevant" to "relevant").

	For my professional practice, the topic is…															For medical studies, the topic is…				
	…challenging					…frequent					…significant									
	very easy				very difficult	very rare				very often	unimportant				very important	irrelevant				relevant
**Initial consultation und medical history**																				
Initial consultation	□	□	□	□	□	○	○	○	○	○	◇	◇	◇	◇	◇	△	△	△	△	△
Medical history	□	□	□	□	□	○	○	○	○	○	◇	◇	◇	◇	◇	△	△	△	△	△
Psychosocial history	□	□	□	□	□	○	○	○	○	○	◇	◇	◇	◇	◇	△	△	△	△	△
Sexual history	□	□	□	□	□	○	○	○	○	○	◇	◇	◇	◇	◇	△	△	△	△	△
Other topic: ____________________																				
**Sensitive Topics**																				
Addressing domestic violence, abuse, or maltreatment	□	□	□	□	□	○	○	○	○	○	◇	◇	◇	◇	◇	△	△	△	△	△
Addressing addiction issues (e.g. addressing alcohol consumption)	□	□	□	□	□	○	○	○	○	○	◇	◇	◇	◇	◇	△	△	△	△	△
Sexuality, sexual dysfunction, and addressing questions about intimate issues	□	□	□	□	□	○	○	○	○	○	◇	◇	◇	◇	◇	△	△	△	△	△
Addressing and managing suicidality	□	□	□	□	□	○	○	○	○	○	◇	◇	◇	◇	◇	△	△	△	△	△
Dealing with death, dying, and grief	□	□	□	□	□	○	○	○	○	○	◇	◇	◇	◇	◇	△	△	△	△	△
Other sensitive topic: ________________________________	□	□	□	□	□	○	○	○	○	○	◇	◇	◇	◇	◇	△	△	△	△	△
**Specific illnesses**																				
Chronic illnesses	□	□	□	□	□	○	○	○	○	○	◇	◇	◇	◇	◇	△	△	△	△	△
Mental illnesses	□	□	□	□	□	○	○	○	○	○	◇	◇	◇	◇	◇	△	△	△	△	△
Malignant diseases	□	□	□	□	□	○	○	○	○	○	◇	◇	◇	◇	◇	△	△	△	△	△
Dementia and Alzheimer's Disease	□	□	□	□	□	○	○	○	○	○	◇	◇	◇	◇	◇	△	△	△	△	△
Other medical conditions: ____________	□	□	□	□	□	○	○	○	○	○	◇	◇	◇	◇	◇	△	△	△	△	△
**Specific situations and contexts**																				
Breaking bad news	□	□	□	□	□	○	○	○	○	○	◇	◇	◇	◇	◇	△	△	△	△	△
Recommendation of behaviour changes (health promotion)	□	□	□	□	□	○	○	○	○	○	◇	◇	◇	◇	◇	△	△	△	△	△
Communication and explanation of risks	□	□	□	□	□	○	○	○	○	○	◇	◇	◇	◇	◇	△	△	△	△	△
Dealing with non-adherent patients (compliance)	□	□	□	□	□	○	○	○	○	○	◇	◇	◇	◇	◇	△	△	△	△	△
Dealing with patients with health anxiety and illness fears	□	□	□	□	□	○	○	○	○	○	◇	◇	◇	◇	◇	△	△	△	△	△
Dealing with time pressure (time management)	□	□	□	□	□	○	○	○	○	○	◇	◇	◇	◇	◇	△	△	△	△	△

	For my professional practice, the topic is…			For medical studies, the topic is…
	…challenging	…frequent	…significant	
	very easy – very difficult	very rare – very often	unimportant – very important	irrelevant – relevant
Communicating one's own mistakes	□ □ □ □ □	○ ○ ○ ○ ○	◇ ◇ ◇ ◇ ◇	△ △ △ △ △
Dealing with uncertainty in medical decisions	□ □ □ □ □	○ ○ ○ ○ ○	◇ ◇ ◇ ◇ ◇	△ △ △ △ △
Dealing with patients' emotions	□ □ □ □ □	○ ○ ○ ○ ○	◇ ◇ ◇ ◇ ◇	△ △ △ △ △
Dealing with children	□ □ □ □ □	○ ○ ○ ○ ○	◇ ◇ ◇ ◇ ◇	△ △ △ △ △
Interprofessional communication	□ □ □ □ □	○ ○ ○ ○ ○	◇ ◇ ◇ ◇ ◇	△ △ △ △ △
Care of palliative patients	□ □ □ □ □	○ ○ ○ ○ ○	◇ ◇ ◇ ◇ ◇	△ △ △ △ △
Psychosomatics	□ □ □ □ □	○ ○ ○ ○ ○	◇ ◇ ◇ ◇ ◇	△ △ △ △ △
Conveying information and confirming understanding	□ □ □ □ □	○ ○ ○ ○ ○	◇ ◇ ◇ ◇ ◇	△ △ △ △ △
Other situation or context: ________________________	□ □ □ □ □	○ ○ ○ ○ ○	◇ ◇ ◇ ◇ ◇	△ △ △ △ △
**Conversations with relatives**				
Conversation with caregivers of a seriously ill child	□ □ □ □ □	○ ○ ○ ○ ○	◇ ◇ ◇ ◇ ◇	△ △ △ △ △
Conversation about organ donation or autopsy	□ □ □ □ □	○ ○ ○ ○ ○	◇ ◇ ◇ ◇ ◇	△ △ △ △ △
Conversation about the death of a relative	□ □ □ □ □	○ ○ ○ ○ ○	◇ ◇ ◇ ◇ ◇	△ △ △ △ △
Other conversational context: ________________________	□ □ □ □ □	○ ○ ○ ○ ○	◇ ◇ ◇ ◇ ◇	△ △ △ △ △
**Factors influencing communication**				
Language barriers	□ □ □ □ □	○ ○ ○ ○ ○	◇ ◇ ◇ ◇ ◇	△ △ △ △ △
Sensory impairments such as hearing loss or visual impairment	□ □ □ □ □	○ ○ ○ ○ ○	◇ ◇ ◇ ◇ ◇	△ △ △ △ △
Education	□ □ □ □ □	○ ○ ○ ○ ○	◇ ◇ ◇ ◇ ◇	△ △ △ △ △
Culture	□ □ □ □ □	○ ○ ○ ○ ○	◇ ◇ ◇ ◇ ◇	△ △ △ △ △
Age	□ □ □ □ □	○ ○ ○ ○ ○	◇ ◇ ◇ ◇ ◇	△ △ △ △ △
Gender	□ □ □ □ □	○ ○ ○ ○ ○	◇ ◇ ◇ ◇ ◇	△ △ △ △ △
Disability	□ □ □ □ □	○ ○ ○ ○ ○	◇ ◇ ◇ ◇ ◇	△ △ △ △ △
Religious and philosophical value and norm systems	□ □ □ □ □	○ ○ ○ ○ ○	◇ ◇ ◇ ◇ ◇	△ △ △ △ △
Other influencing factors: ________________________	□ □ □ □ □	○ ○ ○ ○ ○	◇ ◇ ◇ ◇ ◇	△ △ △ △ △

Please name the three topics from the previously mentioned list that, in your opinion, represent …

…the greatest **challenges**:	…occur most **frequently** in your professional daily routine:	… are most **important** for your professional practice:	… are **crucial for a seminar** on doctor-patient communication in medical school:
1.________________	1.________________	1.________________	1.________________
2.________________	2.________________	2.________________	2.________________
3.________________	3.________________	3.________________	3.________________

(B) Personal Continuing Education Behaviour

Have you previously attended seminars or completed training/continuing education courses that included content on doctor-patient communication??

☐ Yes, namely: ☐ during my studies or ☐ in a training/continuing education course

☐ No

Do you feel adequately prepared for your current role in terms of communication with patients?

☐ Yes

☐ No

Attachment 1 to Laudage F, Kötter T, Wiswede D. *From practice to lecture hall: Optimizing communication courses in medical education.* GMS J Med Educ. 2025;42(4):Doc49. DOI: 10.3205/zma001773

(C) Questions about your professional activity and personal information

Finally, we kindly ask you to answer a few questions about your practice and yourself.

Your age: ☐ Younger than 36 ☐ 36-50 ☐ 51-65 ☐ Older than 65

Your gender: ☐ Female ☐ Male ☐ Other: ____________

What type of practice do you work in?

☐ Single Practice

☐ Group Practice

☐ Medical Facility e.g. CHC

What specialist training have you completed?

☐ General Medicine

☐ Internal Medicine (working as a general practitioner)

☐ General practitioner without specialist training

☐ ________________________

How many years have you been practicing as a general practitioner?

☐ 0-5 years

☐ 6-10 years

☐ More than 10 years

Is there anything else you would like to share with us regarding seminars on doctor-patient communication during medical school or specifically about this questionnaire?

____

____

Please check that you have answered **every** question. Now, please place the questionnaire in the prepaid return envelope and mail it. **Thank you very much for your participation!**

Attachment 1 to Laudage F, Kötter T, Wiswede D. *From practice to lecture hall: Optimizing communication courses in medical education.* GMS J Med Educ. 2025;42(4):Doc48. DOI: 10.3205/zma001773
