# Supplementary material for: From practice to lecture hall: Optimizing communication courses in medical education
Source: GMS J Med Educ. 2025 Sep 15;42(4):Doc49. doi: 10.3205/zma001773 (PMC12527397; doi:10.3205/zma001773)
Supplement: Supplementary material [file JME-42-49-s-002.pdf]

Attachment 2: Supplementary material

Topics ranked by their relevance for a communication course in medical school

Relevance in medical school	*n*	*M* (*SD*)	Frequencies (%)		Assessment
			irrelevant/ rather irrelevant	rather relevant/ relevant	
Initial consultation	46	4.91 (0.29)	0.0	100	
Medical history	46	4.78 (0.47)	0.0	97.8	
Chronic illnesses	44	4.73 (0.54)	0.0	95.5	
Mental illnesses	44	4.66 (0.61)	0.0	93.2	
Recommendation of behaviour changes (health promotion)	46	4.61 (0.75)	2.2	89.1	
Psychosomatics	44	4.59 (0.62)	0.0	93.2	
Addressing and managing suicidality	46	4.57 (0.72)	0.0	87.0	
Communicating one's own mistakes	45	4.56 (0.69)	2.2	93.3	
Breaking bad news	46	4.54 (0.62)	0.0	93.5	
Addressing addiction issues (e.g. addressing alcohol consumption)	46	4.54 (0.66)	0.0	91.3	
Dealing with death, dying, and grief	46	4.54 (0.84)	4.3	86.9	*relevant*
Malignant diseases	44	4.52 (0.66)	0.0	90.9	
Conveying information and confirming understanding	45	4.51 (0.66)	0.0	91.1	
Dealing with uncertainty in medical decisions	44	4.50 (0.70)	2.3	93.2	
Psychosocial history	46	4.50 (0.78)	2.2	86.9	
Dealing with patients' emotions	44	4.43 (0.70)	0.0	88.6	
Dementia and Alzheimer's Disease	44	4.41 (0.79)	2.3	86.3	
Communication and explanation of risks	46	4.39 (0.71)	0.0	87.0	
Dealing with patients with health anxiety and illness fears	45	4.38 (0.91)	4.4	86.7	
Dealing with non-adherent patients (compliance)	46	4.37 (0.88)	2.2	84.8	*rather relevant*
Conversation about the death of a relative	44	4.36 (0.87)	4.5	84.1	
Addressing domestic violence, abuse, or maltreatment	46	4.33 (0.92)	6.5	82.6	

Relevance in medical school	*n*	*M* (*SD*)	Frequencies (%) irrelevant/ rather irrelevant	rather relevant/ relevant	Assessment
Dealing with time pressure (time management)	45	4.31 (0.97)	8.9	88.9	*rather relevant*
Care of palliative patients	44	4.30 (0.88)	4.5	81.8	
Conversation with caregivers of a seriously ill child	43	4.09 (1.06)	7.0	69.7	
Dealing with children	44	4.07 (0.95)	4.5	68.2	
Sexuality, sexual dysfunction, and addressing questions about intimate issues	44	4.02 (0.95)	6.8	70.4	
Conversation about organ donation or autopsy	43	3.93 (1.14)	9.4	65.2	
Interprofessional communication	44	3.91 (1.07)	9.1	56.8	
Education	44	3.89 (1.02)	11.4	65.9	
Culture	44	3.89 (1.04)	11.4	63.7	
Age	43	3.88 (1.05)	14.0	67.5	
Language barriers	45	3.87 (1.14)	8.8	60.0	
Disability	45	3.84 (1.04)	8.9	55.6	
Sexual history	46	3.83 (1.12)	15.2	58.7	
Sensory impairments such as hearing loss or visual impairment	45	3.73 (1.10)	15.6	55.5	
Religious and philosophical value and norm systems	45	3.64 (1.15)	15.5	55.6	
Gender	44	3.55 (1.27)	25.0	52.3	

Note. The listed topics are color-coded according to their level of relevance.

[a] The arithmetic mean is based on a scale from 1 to 5 (1 = irrelevant; 2 = rather irrelevant; 3 = neither; 4 = rather relevant; 5 = relevant).

Topics ranked by degree of challenge

Challenges in daily professional practice	*n*	*M* (*SD*)	Frequencies (%) very easy/ easy	Frequencies (%) difficult/ very difficult	Assessment
Language barriers	45	4.09 (1.02)	8.9	77.8	difficult
Conversation with caregivers of a seriously ill child	43	4.07 (1.06)	9.3	74.4	
Dealing with non-adherent patients (compliance)	46	3.67 (1.12)	13.0	56.6	
Addressing domestic violence, abuse, or maltreatment	46	3.65 (1.27)	19.5	56.5	
Dealing with time pressure (time management)	45	3.60 (1.05)	15.5	62.2	
Breaking bad news	46	3.48 (1.05)	19.6	52.2	*neither*
Sexuality, sexual dysfunction, and addressing questions about intimate issues	45	3.36 (0.91)	13.3	46.7	
Sexual history	41	3.32 (1.06)	22.0	46.3	
Dealing with patients with health anxiety and illness fears	44	3.27 (1.13)	27.2	43.2	
Sensory impairments such as hearing loss or visual impairment	45	3.18 (0.87)	22.2	37.7	
Communicating one's own mistakes	45	3.07 (0.96)	28.8	35.5	
Dementia and Alzheimer’s Disease	46	3.02 (1.16)	37.0	37.0	
Malignant diseases	46	2.98 (1.06)	30.4	28.3	
Care of palliative patients	43	2.98 (1.06)	34.9	32.6	
Mental illnesses	46	2.96 (1.07)	34.8	32.6	
Dealing with uncertainty in medical decisions	44	2.93 (1.07)	38.6	25.0	
Addressing and managing suicidality	46	2.91 (1.11)	34.8	28.3	
Conversation about the death of a relative	45	2.89 (1.15)	40.0	31.1	
Dealing with patients' emotions	45	2.82 (0.91)	33.3	24.4	
Psychosomatics	44	2.82 (1.08)	36.3	27.2	
Addressing addiction issues (e.g. addressing alcohol consumption)	46	2.70 (1.05)	45.6	26.1	
Dealing with death, dying, and grief	45	2.67 (1.02)	46.6	26.7	
Religious and philosophical value and norm systems	45	2.67 (1.04)	44.4	20.0	
Culture	45	2.64 (1.03)	48.9	26.7	
Psychosocial history	45	2.62 (1.11)	48.9	20.0	
Conversation about organ donation or autopsy	43	2.60 (1.03)	48.9	20.9	

Challenges in daily professional practice	*n*	*M* (*SD*)	Frequencies (%)		Assessment
			very easy/ easy	difficult/ very difficult	
Education	45	2.58 (1.06)	46.7	20.2	
Conveying information and confirming understanding	45	2.44 (0.99)	55.6	17.8	
Disability	45	2.36 (0.74)	62.2	6.7	
Recommendation of behaviour changes (health promotion)	46	2.35 (1.06)	65.3	15.2	
Chronic illnesses	46	2.35 (1.08)	58.7	13.0	
Communication and explanation of risks	46	2.33 (0.87)	63.0	10.9	*easy*
Initial consultation	46	2.26 (0.95)	60.9	10.9	
Interprofessional communication	44	2.20 (0.95)	65.9	11.4	
Medical history	46	2.17 (0.95)	69.6	8.7	
Dealing with children	43	2.14 (0.89)	67.5	7.0	
Age	44	1.93 (0.93)	75.0	6.8	
Gender	44	1.70 (0.77)	81.8	0.0	

Note: The listed topics are presented according to the frequency of occurrence.

[a] The arithmetic mean is based on a scale from 1 to 5 (1 = very easy; 2 = easy; 3 = neither; 4 = difficult; 5 = very difficult).

Topics by frequency of occurrence

Frequency of occurrence in professional daily practice	*n*	*M* (*SD*)	Frequencies (%) very rare/ rare	Frequencies (%) often/ very often	Assessment
Chronic illnesses	45	4.62 (0.80)	4.4	95.5	*very often*
Medical history	45	4.60 (0.62)	0.0	93.4	
Recommendation of behaviour changes (health promotion)	46	4.46 (0.78)	2.2	93.3	
Dealing with time pressure (time management)	44	4.34 (0.86)	4.5	84.0	
Initial consultation	45	4.33 (0.85)	4.4	84.4	
Psychosomatics	43	4.21 (0.86)	2.3	76.7	
Communication and explanation of risks	46	4.15 (0.73)	0.0	80.5	
Psychosocial history	45	4.04 (0.88)	4.4	80.0	
Mental illnesses	45	4.04 (0.90)	4.4	77.7	
Dealing with patients with health anxiety and illness fears	44	3.86 (0.85)	4.6	72.8	*often*
Conveying information and confirming understanding	43	3.86 (1.06)	7.0	65.2	
Dealing with patients' emotions	42	3.79 (1.00)	11.9	61.9	
Addressing addiction issues (e.g. addressing alcohol consumption)	46	3.54 (0.75)	6.5	52.2	
Dealing with death, dying, and grief	46	3.52 (0.89)	10.9	54.4	
Age	43	3.49 (1.22)	21.0	51.2	
Education	45	3.38 (0.96)	20.0	46.7	
Dementia and Alzheimer's Disease	44	3.25 (0.99)	25.0	36.3	
Malignant diseases	45	3.20 (0.87)	20.0	31.1	
Language barriers	45	3.20 (1.16)	31.1	37.8	
Conversation about the death of a relative	44	3.18 (1.17)	31.8	43.1	
Interprofessional communication	42	3.17 (0.94)	23.8	28.6	*occasional*
Dealing with uncertainty in medical decisions	41	3.15 (0.91)	29.3	39.0	
Dealing with non-adherent patients (compliance)	46	3.13 (1.11)	32.6	43.5	
Sensory impairments such as hearing loss or visual impairment	45	2.96 (0.98)	31.1	28.8	
Culture	45	2.91 (0.82)	31.1	22.2	
Breaking bad news	46	2.85 (0.76)	32.6	19.6	
Disability	44	2.70 (0.85)	38.6	13.7	
Care of palliative patients	42	2.67 (1.10)	50.0	21.4	
Gender	42	2.67 (1.18)	47.7	21.4	
Addressing and managing suicidality	46	2.61 (0.83)	43.5	13.0	

Frequency of occurrence in professional daily practice	*n*	*M* (*SD*)	Frequencies (%) very rare/ rare	often/ very often	Assessment
Religious and philosophical value and norm systems	44	2.57 (1.09)	43.2	15.9	
Communicating one's own mistakes	42	2.43 (0.86)	54.8	7.2	
Dealing with children	41	2.41 (1.05)	65.8	17.1	
Sexuality, sexual dysfunction, and addressing questions about intimate issues	45	2.24 (0.80)	71.1	8.9	*rare*
Sexual history	45	2.16 (0.77)	75.6	6.7	
Addressing domestic violence, abuse, or maltreatment	45	1.93 (0.86)	80.0	6.7	
Conversation about organ donation or autopsy	43	1.70 (0.86)	79.1	2.3	
Conversation with caregivers of a seriously ill child	42	1.40 (0.83)	90.5	2.4	*very rare*

Note. The listed topics are presented in order of frequency of occurrence.

[a] The arithmetic mean is based on a scale ranging from 1 to 5 (1 = very rare; 2 = rare; 3 = occasional; 4 = often; 5 = very often).
